# Mechanism of Spontaneous Intracerebral Hemorrhage Formation: An Anatomical Specimens-Based Study

**DOI:** 10.1161/STROKEAHA.122.040143

**Published:** 2022-09-08

**Authors:** Radosław Rzepliński, Mikołaj Sługocki, Sylwia Tarka, Michał Tomaszewski, Michał Kucewicz, Krzysztof Karczewski, Paweł Krajewski, Jerzy Małachowski, Bogdan Ciszek

**Affiliations:** Department of Descriptive and Clinical Anatomy (R.R., M.S., B.C.), Medical University of Warsaw, Poland.; First Department of Anesthesiology and Intensive Care (R.R.), Medical University of Warsaw, Poland.; Department of Forensic Medicine (S.T., P.K.), Medical University of Warsaw, Poland.; Department of Pediatric Neurosurgery, Bogdanowicz Memorial Hospital for Children, Warsaw, Poland (M.S., B.C.).; Department of Neuropathology, Institute of Psychiatry and Neurology, Warsaw, Poland (S.T.).; Institute of Mechanics and Computational Engineering, Faculty of Mechanical Engineering (M.T., M.K., J.M.), Military University of Technology, Warsaw, Poland.; Institute of Materials Science and Engineering, Faculty of Advanced Technologies and Chemistry (K.K.), Military University of Technology, Warsaw, Poland.

## Abstract

Despite advances in understanding various risk and prognostic factors, spontaneous intracerebral hemorrhage is connected to very high morbidity and mortality, while the therapy is mainly supportive. Understanding of the pathophysiology of initial hematoma expansion is limited due to insufficient clinical data and lack of a suitable animal model.

**Methods:**

We injected 40 anatomic specimens of the basal ganglia with contrast medium, scanned them with a micro-computed tomography scanner and analyzed the results of radiological studies, direct and histological examinations.

**Results:**

In 9 cases, micro-computed tomography and histological examinations revealed contrast medium extravasations mimicking intracerebral hematomas. The artificial hematomas spread both proximally and distally along the ruptured perforator and its branches in the perivascular spaces and detached the branches from the adjacent neural tissue leading to destruction of the tissue and secondary extravasations. Moreover, some contrast extravasations skipped to the perivascular spaces of unruptured perforators, created further extravasation sites and aggravated the expansion of the artificial hematoma. There was no subarachnoid extension of any artificial hematoma.

**Conclusions:**

We postulate that a forming basal ganglia intracerebral hematoma spreads initially in the perivascular space, detaches the branches from the neural tissue and causes secondary bleeding. It can also skip to the perivascular space of a nearby perforator. The proposed mechanism of hematoma initiation and formation explains extent of damage to the neural tissue, variability of growth in time and space, creation of secondary bleeding sites, and limited usefulness of surgical interventions. The model is reproducible, the extent of the artificial hematoma can be easily controlled, the rupture sites of the perforating arteries can be determined, and preparation of the model does not require specialized, expensive equipment apart from the micro-computed tomography scanner.

As the most prevalent subtype of hemorrhagic stroke, spontaneous intracerebral hemorrhage (sICH) accounts for ≈15% of all strokes and is related to high morbidity and mortality of about 40%.^1,2^ Despite advances in understanding various prognostic and risk factors as well as neural tissue response to the hematoma,^3–5^ the prognosis remains poor, the therapy is mainly supportive and surgical interventions are limited to selected patients with signs of excessive mass effect, herniation, or hydrocephalus.^6–9^ The scientific community is incrementally building knowledge about ICH pathophysiology to select potential treatment targets.

A ruptured cerebral perforator is thought to be the primary source of bleeding.^10,11^ Interestingly, further studies revealed secondary bleeding sites,^12^ and a theory was adapted that a growing spherical hematoma stretches surrounding vessels and makes them rupture.^13–15^ However, currently exploited experimental models are based on intracerebral injection of autologous blood or collagenase, which does not reflect in vivo conditions of hematoma initiation.^16^

In this study, we describe a novel model of basal ganglia ICH initiation based on anatomic specimens and present observations regarding hematoma initiation and formation.

## Methods

The data that support the findings of this study are available from the corresponding author upon reasonable request. We created the presented model of spontaneous basal ganglia ICH as a part of the National Science Center awarded project “Modelling hemodynamics of small diameter cerebral circulation arteries under physiological conditions and after stenting”. The study protocol was approved by the Ethics Committee of Medical University of Warsaw, Poland (Number 20/2021).

We injected intraarterially contrast medium (a mixture of barium sulfate and gelatin) into 40 unfixed anatomic specimens of the basal ganglia, which were subsequently fixed in buffered 10% formalin solution and scanned with a Nikon/Metris XT H 225 ST micro-computed tomography (CT) scanner (for detailed step-by-step description of specimens preparation please refer to our methodology article^17^). Thanks to high resolution (voxel size of up to 27 µm), the method clearly visualizes all perforating arteries branching from the middle cerebral artery (the lenticulostriate arteries).^18^ We also collected autopsy data about age, sex, and presence of atherosclerosis in 3 areas: the coronary arteries, the circle of Willis, and the aorta. The severity of atherosclerosis was classified as no atherosclerosis, atheromas, fibroatheromas, or complicated lesions.

The injection pressures were measured in additional experiments (Supplemental Material). The pressures were ≈60 mm Hg during injection and maximum 260 mm Hg when the contrast solidifies; the values are medically reasonable and >5× lower than the mean pressure needed to cause a rupture of a major intracranial artery.^19,20^

To our surprise, in 9 specimens the middle cerebral artery collapsed immediately after the injection, suggesting contrast leakage. However, as there were no visible contrast extravasations, we added some more contrast and poured cold water over the specimen to speed up the solidification of the medium.

After micro-CT scanning, we discovered that in such cases, the contrast extravasated intracerebrally and formed hematoma-like configurations, which we called baritomas. The micro-CT scans of all such cases were analyzed in Mimics 23.0 (Materialise, NV, Leuven, Belgium) in terms of sources of contrast extravasation and baritoma geometry and spreading. Finally, the specimens were inspected under a microsurgical microscope; we analyzed contrast spreading patterns and identified some of the rupture points. Part of the specimens was used to prepare histological sections (Hematoxylin and Eosin stain, Mallory’s trichrome stain). The groups of specimens with and without contrast extravasations were compared in terms of clinical characteristics by the use of Fisher exact test or Wilcoxon test, as applicable (SAS software, version 9.4, SAS Institute Inc, Cary, NC).

## Results

**Figure 1.**
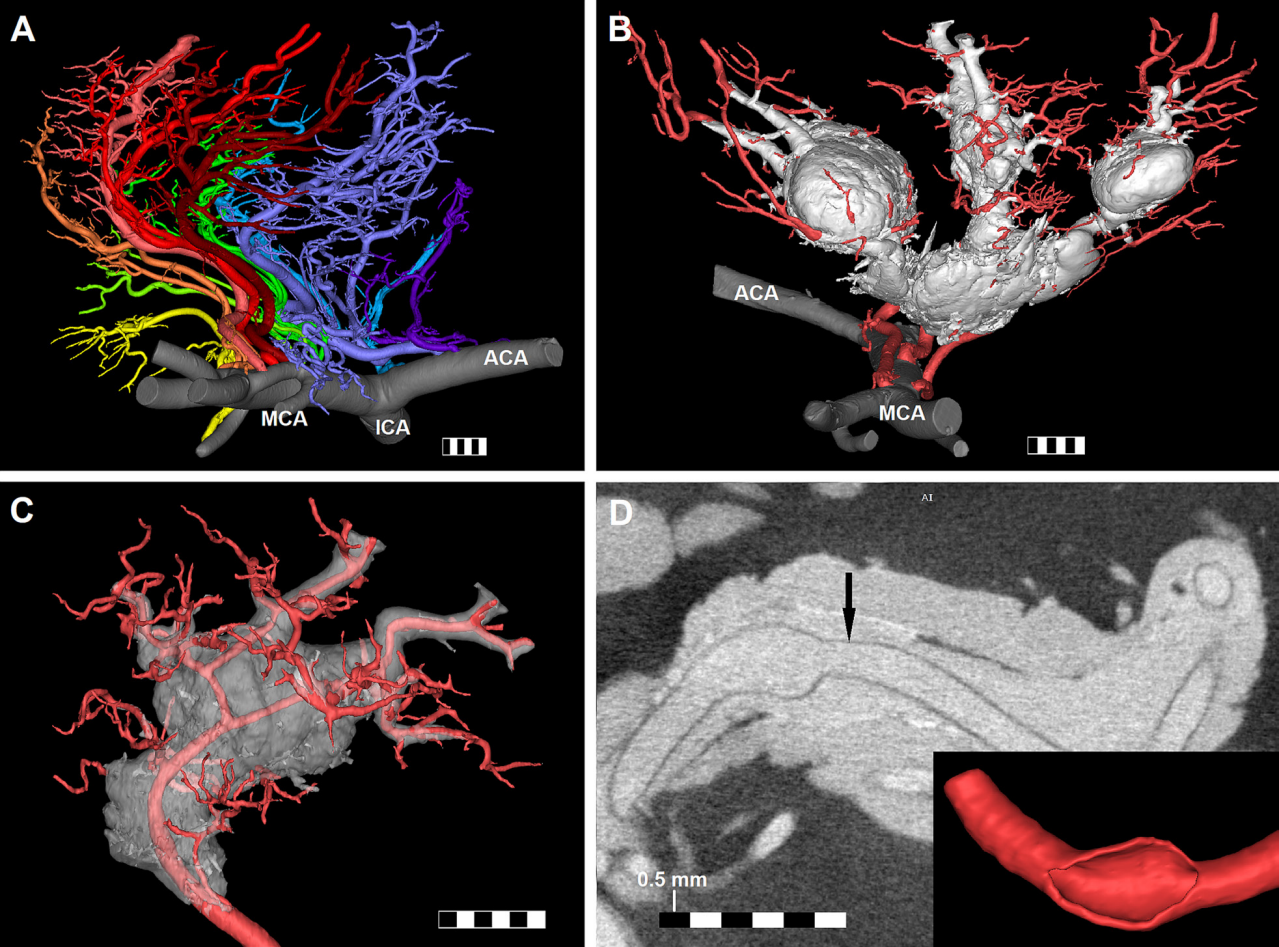
**Results of micro-computed tomography (CT) scanning of the basal ganglia arteries injected with contrast medium. A**, Normal perforating arteries branching from the right middle, anterior, and internal cerebral arteries (MCA, ACA, and ICA) visualized by our method. **B**, Contrast medium extravasations around 3 perforating arteries branching from the left MCA. Note the proximal and distal spread of the artificial hematoma around the parent artery and its branches. **C**, One of the perforators from image **B** with transparent reconstruction of artificial hematoma. Note the lack of visualized branches along the intrahematomal course of the perforator and compare it with image **A**. **D**, Example of perforating artery rupture site: axial image and 3-dimensional reconstruction (inset). High resolution of micro-CT allows to clearly visualize the perforator and its wall (arrow) inside the contrast extravasation. One small segment of every bar is 0.5-mm long; the whole bar is 3-mm long.

We identified contrast medium extravasations in 9 out of 40 cases (4 females and 5 males, age range 21–68 years; Table); all artificial hematomas were located in the basal ganglia. There were no differences in terms of clinical characteristics between the groups of specimens with and without contrast extravasations. In 7 cases (77%), we were able to identify rupture sites in the walls of perforating arteries (Figure 1), which we divided into 2 types: longitudinal, measuring a few millimeters rupture of a major penetrating artery (Figure 1D) or an arteriole ending abruptly in the baritoma; the ruptured arteries had diameters between 0.11 and 0.7 mm. There were multiple rupture sites in 5 cases (55%). In all cases, the contrast medium spread both distally and proximally along the lacerated perforating artery and its branches, forming a cuff around the arterial tree, which was the widest in the proximity of the rupture point (Figure 1B and 1C). In 7 cases (77%), we identified small distal branches of a ruptured perforating artery that ended in the baritoma. We identified 3 cases (33%), where the contrast medium formed a cuff around an artery without visible rupture sites; in all such cases the resulting baritoma was continuous with another one.

**Table.**
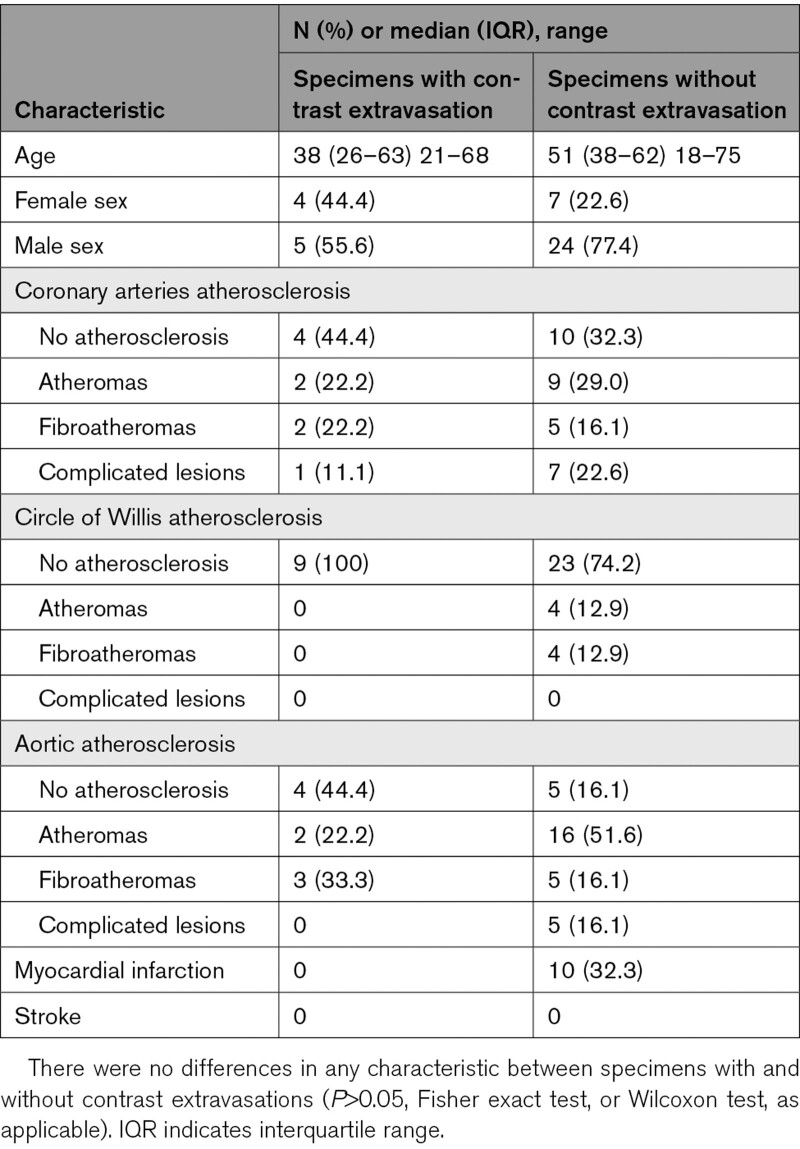
Characteristics of Studied Group

**Figure 2.**
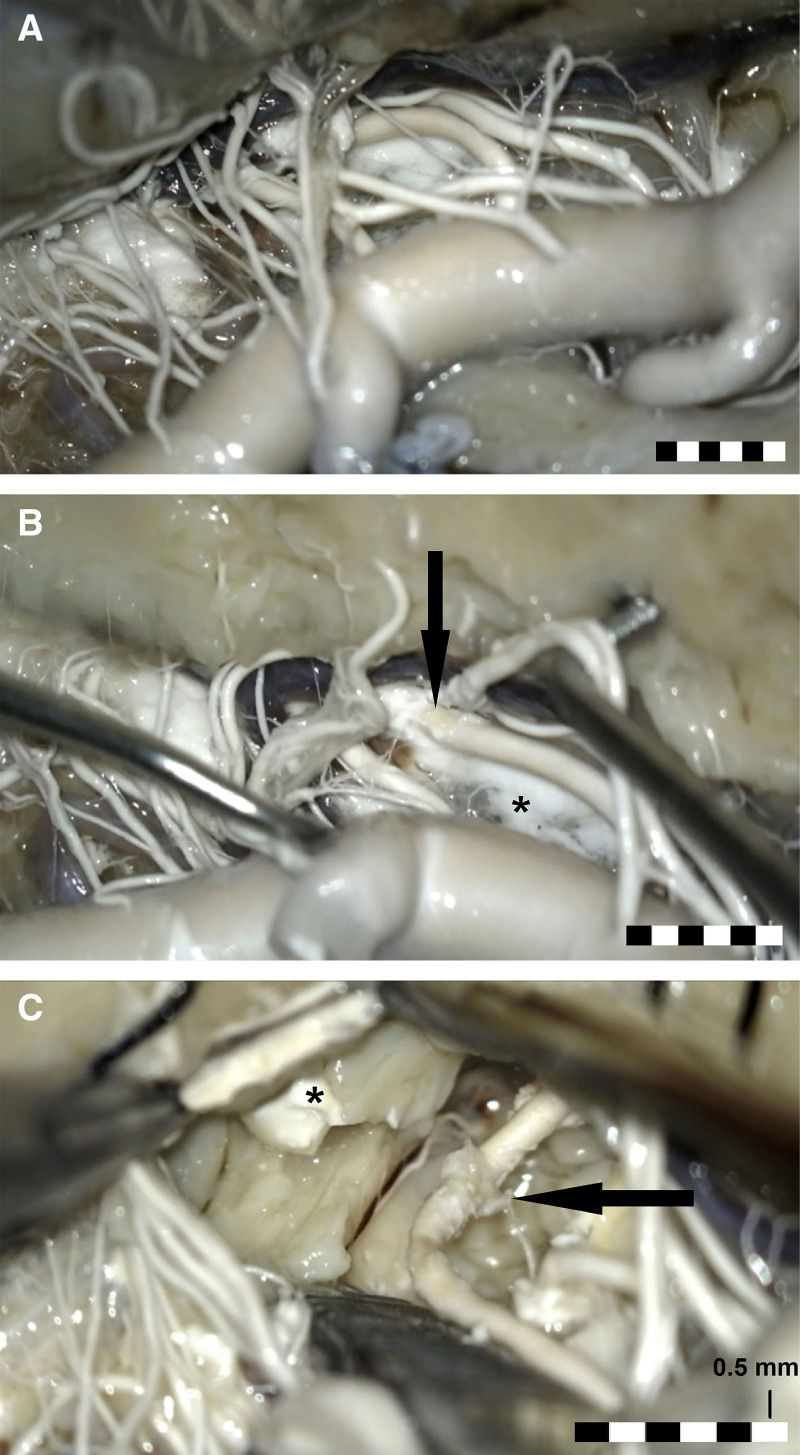
**Microscopical examinations. A**, The middle cerebral artery (MCA) and its perforating branches to the anterior perforated substance. The temporal lobe was lifted with a spatula. **B**, Taking advantage of the results of micro-computed tomography scanning, extracerebral course of the ruptured perforating artery (Figure 1D) was identified and the place, where it digs into the anterior perforated substance was visualized (arrow). Please note the subpial extension of the artificial hematoma (asterisk). **C**, The perforator was followed intracerebrally and the extravasated contrast medium was partially removed. The rupture site reconstructed in Figure 1D was on the posterior wall of the artery and is not visible here. Please note the torn arterial wall (arrow) and the cavity of artificial hematoma and contrast medium (asterisk). One small segment of every bar is 0.5-mm long; the whole bar is 3-mm long.

Microscopical examinations revealed that 5 baritomas (55%) expanded superficially to the surface of the anterior perforated substance (Figure 2). Noteworthy, we did not find the contrast neither in the subarachnoid space nor around the arteries in the subarachnoid space. Further dissection revealed contrast medium intracerebrally and some rupture sites (Figure 2C). Small branches encased in the baritoma were detached from the neural tissue, as visualized by micro-CT.

**Figure 3.**
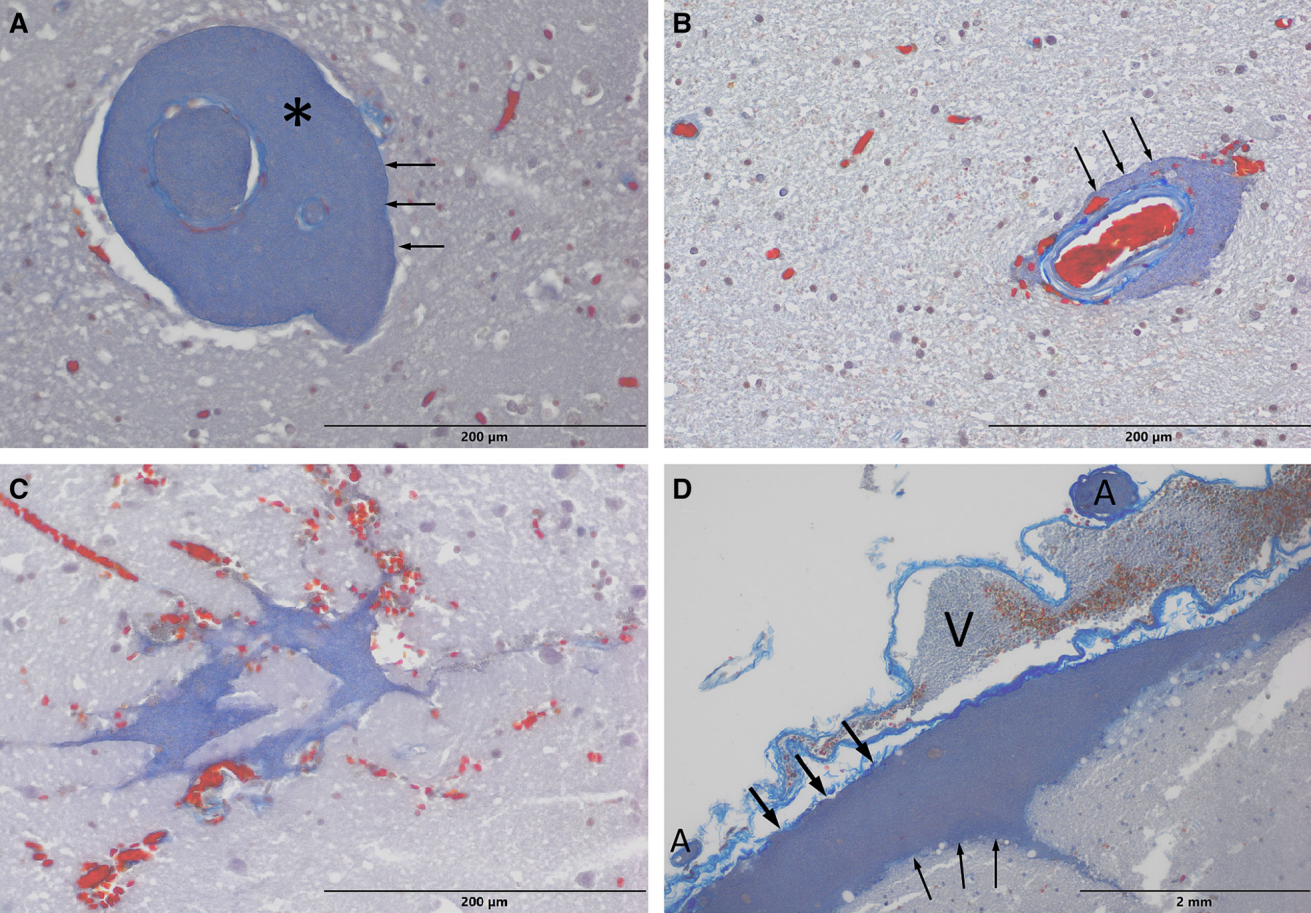
**Histological examinations (Mallory’s trichrome stain). A**, Small penetrating artery and its branch filled with and surrounded by the contrast medium (asterisk) extravasated to the perivascular space. The perivascular space is separated from the parenchyma by the basement membrane (arrows). **B**, Another example of the contrast medium extravasation to the perivascular space. Although the penetrating artery did not fill with contrast due to the presence of the blood clot, the contrast medium reached its perivascular space. The basement membrane is marked with arrows. **C**, The contrast medium extravasated intracerebrally, outside the perivascular space. The neural tissue is mixed with blood clots. **D**, Cross section through the anterior perforated substance. The contrast medium is present in the subpial space, between the pia mater (solid arrows) and the glia limitans (thin arrows). The deep middle cerebral vein (V) and branches of the middle cerebral artery (A) filled with the contrast medium are visible in the subarachnoid space.

The histological examination revealed intracerebral contrast medium extravasations: in some places the contrast medium formed a round cuff around the penetrating arteries and the surface of adjacent neural tissue (the glia limitans) was intact (Figure 3A and 3B); in other places the contrast was present inside destroyed neural tissue (Figure 3C). In cases, where the contrast spread proximally to the anterior perforated substance, presence of the contrast medium under the pia mater in the subpial space was confirmed (Figure 3D).

## Discussion

Artificial hematomas (so-called baritomas) have the typical histology, localization, and bleeding sources of basal ganglia ICH,^21–23^ the contrast medium can spread in unfixed human brain specimens, and its solidification may mimic clotting process; therefore, the presented method allows for the study of sICH initiation and formation. The extent of the baritoma can be controlled by changing the injection pressure and duration (number of rupture points and volume of artificial hematoma) as well as the contrast and specimen temperatures (speed of contrast medium solidification—volume of artificial hematoma). Micro-CT scanning provides excellent data on baritoma spread and rupture sites, leaving the specimen intact, allowing for further investigations (eg, histological studies). The model is unfixed human brain specimens-based, reproducible, and its preparation does not require specialized, expensive equipment or reagents apart from the micro-CT scanner and the microsurgical microscope. As opposed to collagenase and injection models of sICH,^16^ our model is suitable for studying the initial phase of intracerebral bleeding.

The studied group involved both females and males of different ages. Importantly, the study was not designed to analyze influence of different factors on presence of contrast extravasations and drawing such inferences would be misleading. Another study is needed to access the tendency of the perforating arteries to rupture and cause intracerebral bleeding in different populations. Collecting such data will also guide choice of dedicated injection pressures for different specimens. By the time, it can be expected that increasing the injection pressure will lead to increased model reproducibility.

**Figure 4.**
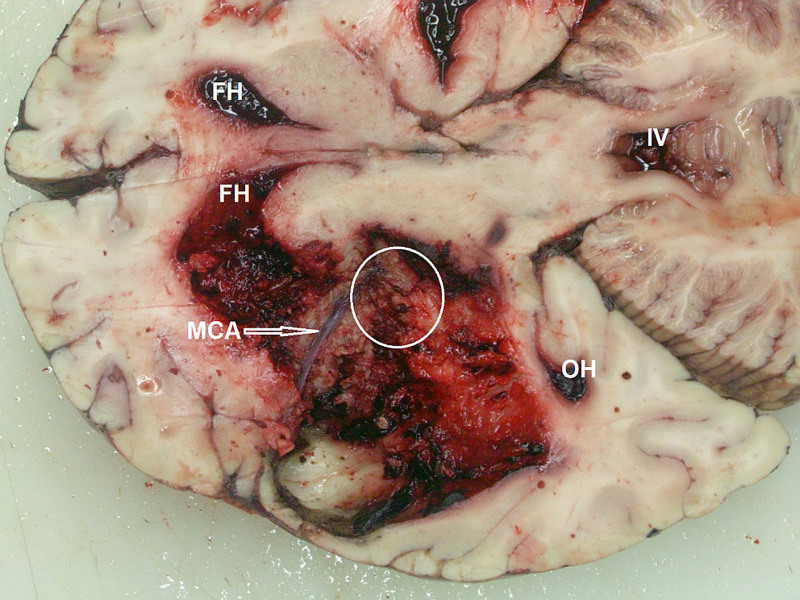
**Illustrative case of a fatal spontaneous intracerebral hemorrhage to the left cerebral hemisphere (top view).** Blood clots were removed from the left basal ganglia and the left middle cerebral artery (MCA) was exposed. Deep cerebral perforators were encased in the hematoma (circle). The hematoma extended to the ventricular system: the frontal (FH) and occipital horns (OH) of the lateral ventricles and the fourth ventricle (IV). Material of the Department of Forensic Medicine, Medical University of Warsaw, Warsaw, Poland.

Our results explain some of the properties of sICH. We found that contrast medium spread both proximally and distally along the ruptured perforating artery and its branches and detached them from the neural tissue, therefore, creating secondary extravasation sites. The fluid-filled perivascular space may serve as a highway for the forming hematoma. As its capacity is limited—the perivascular space disappears at the level of precapillary arteries^24,25^—once filled with blood it may detach arterioles from the tissue, interrupting the blood supply, irreversibly destroying the structure of adjacent neural tissue and causing secondary bleeding. Moreover, we observed some baritomas without visible extravasation points but communicating with one another, which suggests that bleeding can skip to the perivascular space of an unruptured perforator, detach it from the tissue, enlarge the volume of destroyed neural tissue and create further bleeding sites, which closes the vicious circle. Secondary bleeding from the arterioles located in the periphery of the growing hematoma is a well-known neuropathological phenomenon, similarly to the presence of perforators inside the hematoma^21,26^ (see also Figure 4).

As shown in the animation (Video S1), the initial pattern of hematoma expansion may be different from a growing ball: the hematoma not only compresses the adjacent tissue, but above all penetrates and destroys the compliant, healthy tissue using the perivascular spaces as highways. After their filling and creating secondary bleeding points, the pattern of expansion may mimic growing ball. Consequently, sICH growth varies in space and time, and causes extensive damage, as blood clots are partially mixed with gray and white matter. This phenomenon explains the extremely high morbidity and mortality, as well as limited usefulness of sICH surgical removal—during surgical intervention neural tissue is removed with blood clots; it alleviates the secondary brain injury by, among others, reducing the intracranial pressure, but the supply areas of affected perforators remain irreversibly damaged. It is worth recalling that the deep cerebral perforating arteries supply vital neural structures (eg, the internal capsule).^27–31^ Invasive surgical interventions may also lead to further damage, therefore, minimally invasive approaches should be preferred.^9^

It is sometimes believed that the perivascular space is freely connected with the subarachnoid space. However, we observed extravasations of the contrast to the subpial space but not to the subarachnoid space. Similarly, anatomic studies revealed membranes separating subarachnoid and perivascular spaces as well as described differences in the perivascular spaces structure of the deep and cortical perforating arteries.^25,32^ This phenomenon is reflected in the fact that subarachnoid extension of sICH is an uncommon but serious complication of sICH.^33^ This suggests that in the case of basal ganglia perforators, high pressure is needed to create communication between the subpial and subarachnoid spaces. The relationship between perivascular spaces of deep cerebral perforators, and subpial and subarachnoid spaces awaits further study, as most of the studies do not distinguish between the deep and cortical perforating arteries and are based on animal models. Another question is whether blood clots that surround the ruptured perforating artery can cause a platelet response similar to that observed in the case of subarachnoid hemorrhage.^34,35^

Our method also has some limitations. First, it is only a model of sICH and the results were obtained artificially (however, it is not an animal model). Of note, Haider et al. showed that Virchow-Robin spaces usually do not change after death and formalin fixation.^36^ Second, the response to and evolution of ICH cannot be studied. Third, the sample size was not large enough to study the relationships between various patient-specific factors (age, sex, history of hypertension, etc) and hematoma initiation and formation. Additionally, taking into account the model reproducibility of about 25%, dedicated injection pressures will be able to be determined after measuring perforating arteries rupture pressures in another study.

## Article Information

### Acknowledgments

The authors sincerely thank those who donated their bodies to Science so that anatomic research could be performed. The results from such research can potentially increase mankind’s overall knowledge that can then improve patient care. Therefore, these donors and their families deserve our highest gratitude. All procedures performed in the study were in accordance with the ethical standards of the institutional research committee and with the 1964 Helsinki Declaration and its later amendments. The study protocol was approved by The Ethics Committee of Medical University of Warsaw, Poland (Number 20/2021).

### Sources of Funding

The study was funded by the National Science Center, Poland (award number 2020/37/B/ST8/03430, Recipient: Dr Małachowski). The National Science Center had no involvement in the study design, in the collection, analysis and interpretation of data, in the writing of the article, or in the decision to submit the article for publication. Dr Tomaszewski is a recipient of the Foundation for Polish Science scholarship.

### Disclosures

None.

### Supplemental Material

Supplemental Methods

Preclinical Checklist

Video S1

## Supplementary Material


