# Supplementary material for: Mechanism of Spontaneous Intracerebral Hemorrhage Formation: An Anatomical Specimens-Based Study
Source: Stroke. 2022 Sep 8;53(11):3474–80. doi: 10.1161/STROKEAHA.122.040143 (PMC9586829; doi:10.1161/STROKEAHA.122.040143)
Supplement: Supplementary file 1 [file str-53-3474-s001.pdf]

SUPPLEMENTAL MATERIAL

Mechanism of spontaneous intracerebral hemorrhage formation: an anatomical specimens-based study

Stroke 2022

Radosław Rzepliński, Mikołaj Sługocki, Sylwia Tarka, Michał Tomaszewski, Michał Kucewicz, Krzysztof Karczewski, Paweł Krajewski, Jerzy Małachowski, Bogdan Ciszek

Corresponding author: Radosław Rzepliński, radoslaw.rzeplinski@wum.edu.pl
Department of Descriptive and Clinical Anatomy, Medical University of Warsaw, Warsaw, Poland.

Quantification of contrast medium injection pressure

One of the steps of preparing the method of creating 3-dimensional models of cerebral perforating arteries based on anatomical specimens was quantification of contrast medium injection pressure. The injection process is dynamic, the contrast medium solidifies in a few seconds, therefore direct pressure measurement is unreliable. As pressure in the injected arteries cannot exceed the pressure in the syringe, we decided to measure the pressure generated in the syringe in an experimental model. We used a standard system dedicated for invasive arterial blood pressure monitoring, connected it to the same cannula used for injecting the specimens (shortened intravenous cannula with the diameter of 2.1 mm) and performed a series of experiments (Fig. S1).

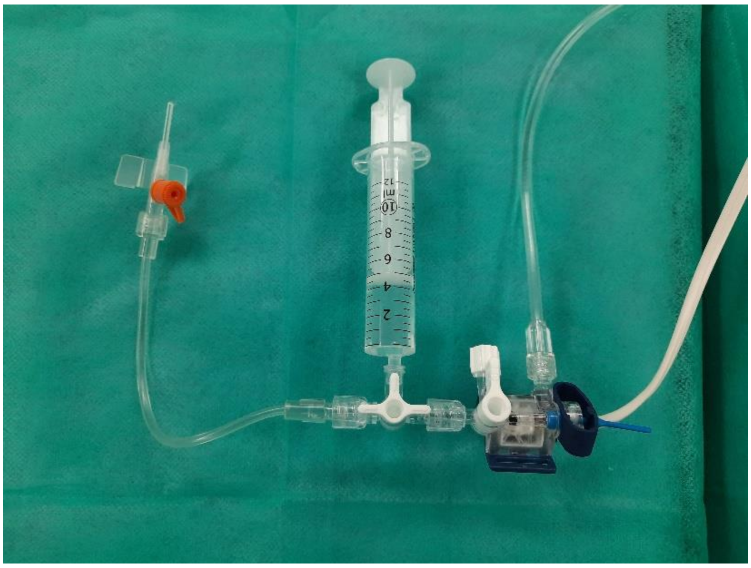

Fig. S1 Experimental setting used for the measurement of injection pressures. A system dedicated for invasive blood pressure monitoring was connected to a cannula used for specimen injecting.

The measurements were taken in two situations: (1) fluid administration with a velocity of 5 ml/s and 10 ml/s and (2) applying the same pressure to the syringe plunger with a closed stopcock as during specimen preparation. The second case simulates the situation, when the contrast medium fills the arteries, starts to solidify, and flow ceases. The experiment was performed by the researcher, who injected the specimens (RR). The measurements were taken 10 times in each situation to limit the effect of subjectivity. The results are presented in Table S1.

Table S1. Pressures measured during the experiments.

	injection 5 ml/s	injection 10 ml/s	closed stopcock
median pressure (Q1-Q3) [mmHg]	53.5<br>(45.5 – 62.5)	165<br>(155.5 – 198.5)	260<br>(215.5 – 287)

Q1 first quartile, Q3 third quartile

Our experiment suggests that during injecting the specimens with contrast medium the pressures are physiological. As measured in the second situation, the critical phase is when the contrast starts to solidify: the pressure has to be maintained to prevent collapsing of the arteries when the contrast penetrates the microcirculation but applying an uncontrolled force to the syringe plunger may lead to generating very high pressures (above 300 mmHg). Therefore, it is essential that the specimens have to be prepared by the same, experienced person. However, a pressure of about 250mmHg can be easily observed in hypertensive patients on a daily basis (e.g., during Valsalva maneuver or lifting heavy objects) and even the pressure of about 300 mmHg is more than 5 times smaller than the average pressure needed to cause a rupture of a major intracranial artery [19]. We can conclude that the pressures generated during specimens' preparation are medically reasonable.

Preclinical Checklist based on:

Reporting Standards for Preclinical Studies of Stroke Therapy
Farhaan Vahidy et al.
https://doi.org/10.1161/STROKEAHA.116.013643 Stroke. 2016;47:2435–2438

Table 1. Checklist of Methodological and Reporting Aspects for Articles Submitted to Stroke Involving Preclinical Experimentation

Methodological and Reporting Aspects	Description of Procedures	Author Comments
Experimental groups and study timeline	□ The experimental group(s) have been clearly defined in the article, including number of animals in each experimental arm of the study.<br>□ An account of the control group is provided, and number of animals in the control group has been reported. If no controls were used, the rationale has been stated.<br>□ An overall study timeline is provided.	Not applicable.<br>Not applicable.<br>Not applicable.
Inclusion and exclusion criteria	□ A priori inclusion and exclusion criteria for tested animals were defined and have been reported in the article.	Not applicable.
Randomization	□ Animals were randomly assigned to the experimental groups. If the work being submitted does not contain multiple experimental groups, or if random assignment was not used, adequate explanations have been provided.<br>□ Type and methods of randomization have been described.<br>□ Methods used for allocation concealment have been reported.	Not applicable.<br>Not applicable.<br>Not applicable.
Blinding	□ Blinding procedures have been described with regard to masking of group/treatment assignment from the experimenter. The rationale for nonblinding of the experimenter has been provided, if such was not feasible.<br>□ Blinding procedures have been described with regard to masking of group assignment during outcome assessment.	Not applicable.<br>Not applicable.
Sample size and power calculations	□ Formal sample size and power calculations were conducted based on a priori determined outcome(s) and treatment effect, and the data have been reported. OR A formal size assessment was not conducted and a rationale has been provided.	Not applicable.

Data reporting and statistical methods	□ Number of animals in each group: randomized, tested, lost to follow-up, or died have been reported. If the experimentation involves repeated measurements, the number of animals assessed at each time point is provided, for all experimental groups.	Not applicable.
	□ Baseline data on assessed outcome(s) for all experimental groups have been reported.	Not applicable.
	□ Details on important adverse events and death of animals during the course of experimentation have been provided, for all experimental arms.	
	□ Statistical methods used have been reported.	Yes.
	□ Numeric data on outcomes have been provided in text, or in a tabular format with the main article or as supplementary tables, in addition to the figures.	Not applicable.
Experimental details, ethics, and funding statements	□ Details on experimentation including stroke model, formulation and dosage of therapeutic agent, site and route of administration, use of anesthesia and analgesia, temperature control during experimentation, and postprocedural monitoring have been described.	Yes.
	□ Different sex animals have been used. If not, the reason/justification is provided.	Not applicable.
	□ Statements on approval by ethics boards and ethical conduct of studies have been provided.	Yes.
	□ Statements on funding and conflicts of interests have been provided.	Yes.
